# Exploring Key Genes and Pathways of Cardiac Hypertrophy Based on Bioinformatics

**DOI:** 10.1155/2022/2081590

**Published:** 2022-08-22

**Authors:** Zhenzhen Zhang, Chunxiao Wang

**Affiliations:** Department of Cardiology, The Affiliated Yantai Yuhuangding Hospital of Qingdao University, Yantai 264000, China

## Abstract

**Objective:**

This research is aimed at identifying the key genes and pathways of cardiac hypertrophy using bioinformatics and at providing a new target for the identification of cardiac hypertrophy.

**Methods:**

Microarray data GSE1621 and GSE18801 were acquired from the GEO database. The DEGs of GSE1621 and GSE18801 were analyzed using the online tool GEO2R. “ggplot2” package of R software was utilized to generate the volcano plots. The top and bottom 10 genes were mapped as a heat map. GO functional annotation analysis and KEGG pathway enrichment analysis were performed separately for DEGs using the online software DAVID. Histograms were plotted using the R “ggplot2” package. The DEGs were imported into the STRING online database for constructing PPI networks and analyzing the DEG interaction relationships.

**Results:**

In the present study, 469 DEGs were screened in GSE1621 and a total of 793 DEGs were screened in GSE18801. GO analyses indicate that DEGs were mainly involved in cardiac muscle contraction, regulation of blood circulation, regulation of muscle contraction, muscle contraction, striated muscle contraction, regulation of heart contraction, regulation of striated muscle contraction, and tissue remodeling. KEGG analyses indicate that DEGs were mainly involved in Th17 cell differentiation, Th1 and Th2 cell differentiation, HIF-1 signaling pathway, pathways in cancer, hematopoietic cell lineage, Chagas disease and cell adhesion molecules, viral myocarditis, central carbon metabolism in cancer, acute myeloid leukemia, and JAK-STAT signaling pathway. Eight hub genes were screened, including Akt1, Lox, Timp1, Col1al, Spp1, Ccnd1, Mmp3, and Egfr.

**Conclusions:**

The DEGs associated with cardiac hypertrophy were screened via bioinformatics analysis, and eight hub genes were identified, including Akt1, Lox, Timp1, Col1al, Spp1, Ccnd1, Mmp3, and Egfr, which might be a new target for the identification of cardiac hypertrophy.

## 1. Introduction

Cardiac hypertrophy is an adaptive response of the myocardium to increased sustained load and often leads to cardiovascular disease [1]. Patients with cardiac hypertrophy clinically show fatigue, dizziness, chest pain, and dyspnea, which seriously harm human health [2]. In developing countries, cardiac hypertrophy ultimately induced heart failure for approximately 25% of all deaths in the population [3, 4].

Cardiac hypertrophy is mainly characterized by hypertrophy of cardiomyocytes and alterations in interstitial components [1]. Pathological myocardial hypertrophy is characterized by protein accumulation, new formation of myogenic fibers, increased expression of embryonic genes, proliferation of myocardial interstitial cells, an increase in the size of cardiomyocytes, and proliferation of connective tissue such as collagen [5, 6]. These pathological changes cause structural disorders of the myocardium, reduced contractility, impaired blood supply, and increased oxygen consumption, resulting in cardiac systolic and diastolic insufficiency, leading to heart failure, arrhythmias, and sudden death. Many cardiovascular diseases such as hypertension, myocardial infarction, heart valve disease, and hyperthyroidism can trigger myocardial hypertrophy, which is mostly considered to be irreversible [5]. The pathogenesis of pathological cardiac hypertrophy is complex and is regulated by various cellular signals [7]. For half a century, although cardiac hypertrophy has been extensively studied at the global, cellular, and molecular levels, the formation process of cardiac hypertrophy remains a mystery.

With the development of bioinformatics, genomics, transcriptomics, proteomics, and metabolomics have generated big data, which can be analyzed by combining bioinformatics and computer science, providing new methods to study the molecular mechanisms of diseases [8]. With the rapid development of gene chips, screening differentially expressed genes (DEGs) and analyzing their functions based on gene chips are currently a new and effective method to study the molecular mechanisms of disease development [9]. Meng et al. obtained GSE129090 dataset and found Bcl-2 participate in progression of cardiac hypertrophy [10]. Katja et al. constructed left ventricular hypertrophy model and screened Efcab6 as new potential candidate gene in left ventricular hypertrophy [11]. The present study is aimed at screening hub genes that participate in cardiac hypertrophy based on GEO database.

In this study, the gene microarray of myocardial hypertrophy in GEO database was used to screen out DEGs, and online databases such as GO and KEGG were utilized to annotate differential gene functions and analyze gene enriched signaling pathways, which provided ideas for identifying new biomarkers of myocardial hypertrophy and revealing potential mechanisms of the disease.

## 2. Material and Methods

### 2.1. Data Collection

Microarray data GSE1621 and GSE18801 were downloaded from the GEO database. GSE1621 included 4 myocardial hypertrophy mice caused by coarctation of aortic arch and 3 sham mice, and the platform was Affymetrix Murine Genome U74A Version 2 Array. GSE18801 included 3 isoproterenol-induced cardiac hypertrophy subjects and 3 normal subjects, and the platform was Affymetrix Mouse Genome 430 2.0 Array.

### 2.2. Identification of DEGs

The DEGs of GSE1621 and GSE18801 were analyzed using the online tool GEO2R. The screening criteria for DEGs were adjusted *P* value < 0.05 and |Log_2_ fold change (LogFC)| >1.5. “ggplot2” package was utilized to generate the volcano plots. The top and bottom 10 genes were mapped as a heat map.

### 2.3. Functional and Pathway Enrichment Analyses

GO functional annotation analysis and KEGG pathway enrichment analysis were performed separately for DEGs using the online software DAVID (https://david.ncifcrf.gov/tools.jsp). Histograms were plotted using the R “ggplot2” package.

### 2.4. PPI Network Construction

The DEGs were first imported into the STRING online database (https://cn.string-db.org/) for analysis. Cytoscape software was used to construct PPI networks and analyze the DEG interaction relationships. Finally, the “MCC” algorithm of “Cytohubba” plugin was used to build the module and screen the candidate hub genes.

## 3. Results

### 3.1. Identification of DEGs

**Figure 1:**
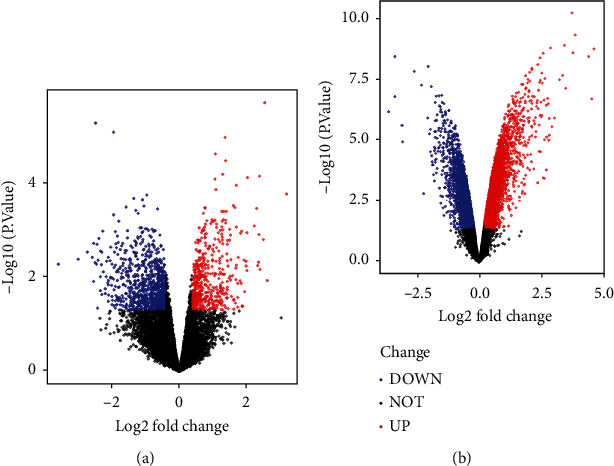
Volcano plots of DEGs in (a) GSE1621 and (b) GSE18801.

**Figure 2:**
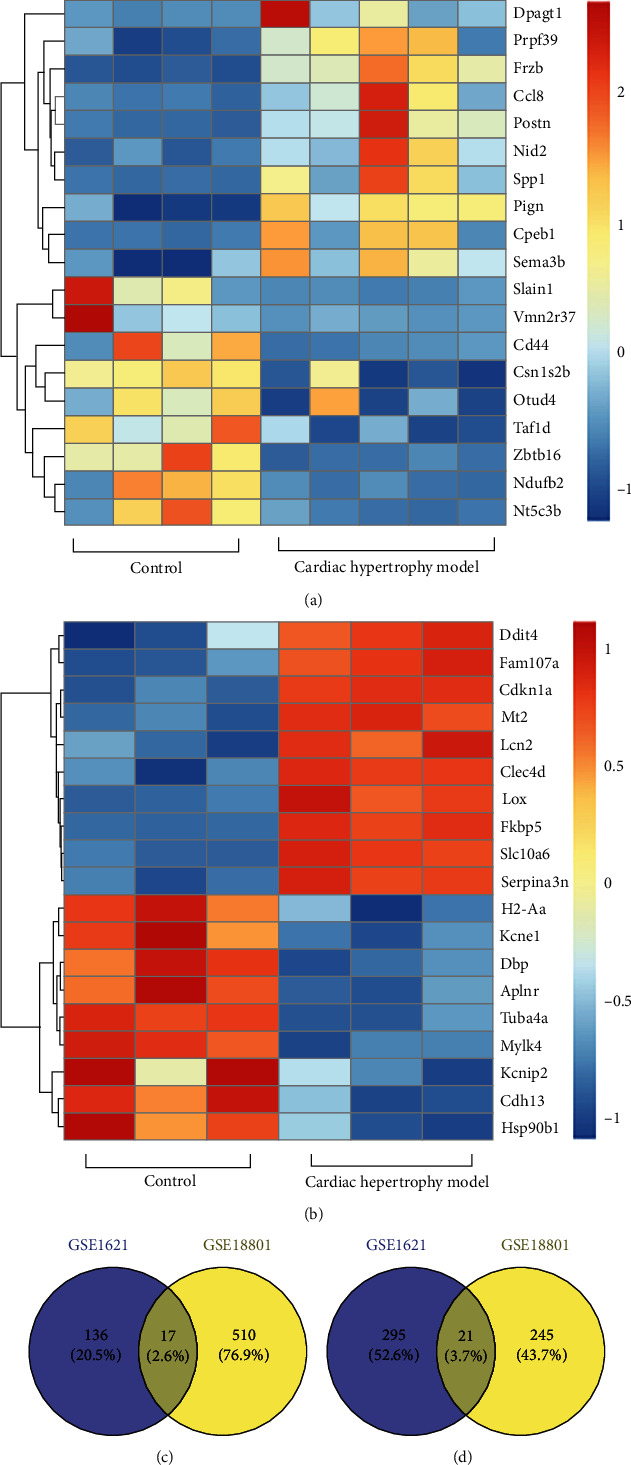
The heat map plots of the top 10 genes and Venn diagrams. (a) The heat map plots of GSE1621. (b) The heat map plots of GSE18801. Venn diagrams illustrated the number of (c) up- and (d) downregulated genes in both datasets, respectively.

153 upregulated DEGs and 316 downregulated DEGs were screened in GSE1621 in the heart tissues of mouse with cardiac hypertrophy (Figure 1(a)). 527 upregulated DEGs and 266 downregulated DEGs were screened in GSE18801 (Figure 1(b)). 38 co-DEGs, including 17 upregulated genes (Figure 2(c)) and 21 downregulated genes (Figure 2(d)), were obtained by taking the intersection from DEGs of GSE1621 and GSE18801. The heat map was generated based on the top and bottom 10 DEGs (Figures 2(a) and 2(b)).

### 3.2. GO Annotation Analyses of DEGs

**Figure 3:**
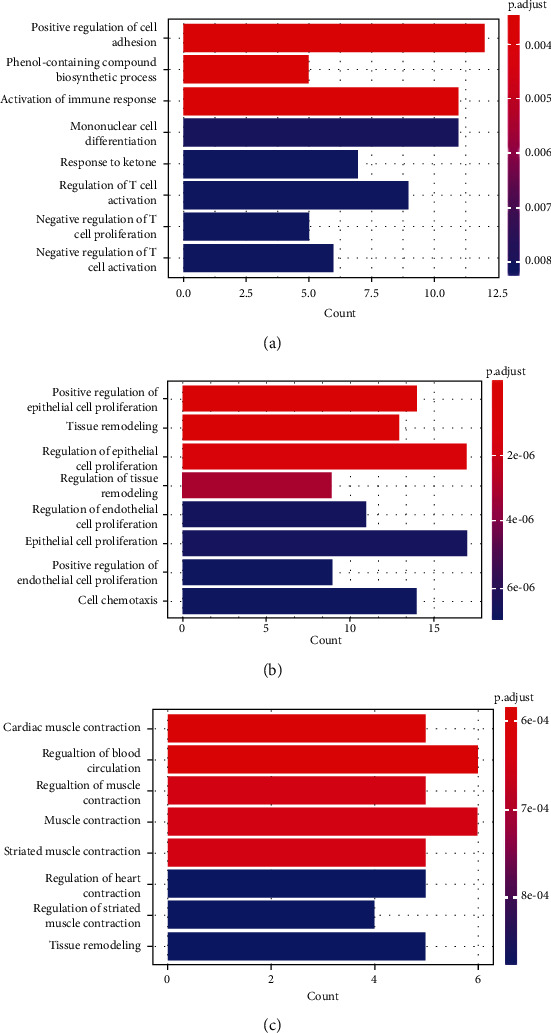
Gene Ontology (GO) analyses of DEGs in (a) GSE1621, (b) GSE18801, and (c) co-DEGs.

The DEGs of GSE1621 participated in positive regulation of cell adhesion, phenol-containing compound biosynthetic process, activation of immune response, mononuclear cell differentiation, response to ketone, regulation of T cell activation, and negative regulation of T cell proliferation and activation (Figure 3(a)). The DEGs of GSE18801 participated in positive regulation of epithelial cell proliferation, tissue remodeling, regulation of epithelial cell proliferation, regulation of tissue remodeling, regulation of endothelial cell proliferation, epithelial cell proliferation, positive regulation of epithelial cell proliferation, and cell chemotaxis (Figure 3(b)). The co-DEGs participated in cardiac muscle contraction, regulation of blood circulation, regulation of muscle contraction, muscle contraction, striated muscle contraction, regulation of heart contraction, regulation of striated muscle contraction, and tissue remodeling (Figure 3(c)).

### 3.3. KEGG Pathway Enrichment Analyses of DEGs

**Figure 4:**
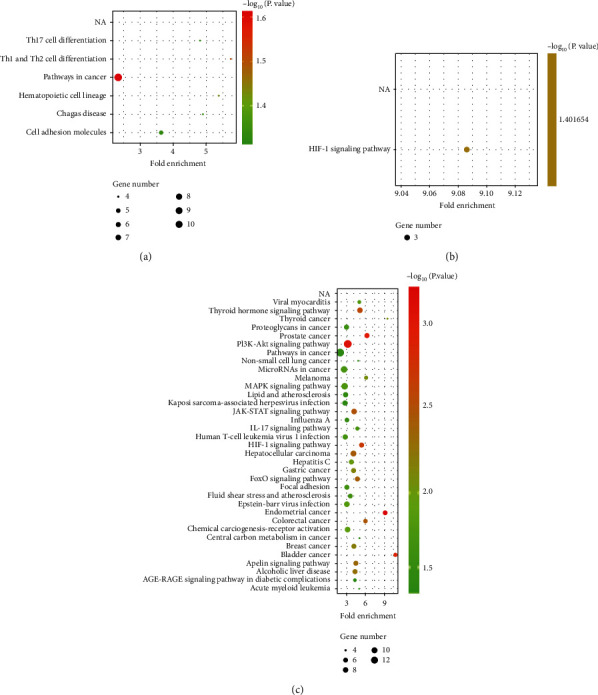
KEGG analyses of DEGs. KEGG analysis of DEGs in (a) GSE1621, (b) GSE18801, and (c) co-DEGs.

The DEGs of GSE1621 were related to Th17 cell differentiation, pathways in cancer, hematopoietic cell lineage, Chagas disease and cell adhesion molecules, and Th1 and Th2 cell differentiation (Figure 4(a)). The DEGs of GSE18801 were related to viral myocarditis, thyroid hormone signaling pathway, FoxO signaling pathway, JAK-STAT signaling pathway, apelin signaling pathway, thyroid cancer, focal adhesion, HIF-1 signaling pathway, chemical carcinogenesis-receptor activation, proteoglycans in cancer, Epstein-Barr virus infection, influenza A, alcoholic liver disease, prostate cancer, lipid and atherosclerosis, IL-17 signaling pathway, PI3K-Akt signaling pathway, MAPK signaling pathway, human T cell leukemia virus 1 infection, pathways in cancer, hepatocellular carcinoma, hepatitis C, melanoma, fluid shear stress and atherosclerosis, and acute myeloid leukemia (Figure 4(b)). The co-DEGs of GSE1621 and GSE18801 were related to the HIF-1 signaling pathway (Figure 4(c)).

### 3.4. PPI Network and Hub Genes

**Figure 5:**
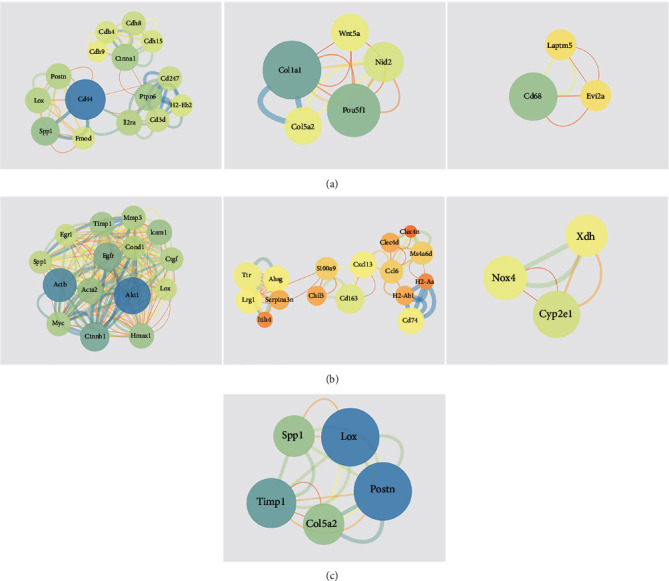
PPI networks based on the screened DEGs in (a) GSE1621, (b) GSE18801, and (c) co-DEGs.

DEGs were uploaded to STRING online software, and PPI network was constructed. The module analysis of GSE1621 using the MCODE plugin found three functional modules (Figure 5(a)), and Col1al was identified as hub genes. The module analysis of GSE18801 using the MCODE plugin found three functional modules (Figure 5(b)), and Ccnd1, Mmp3, Egfr, and Akt1 were identified as hub genes. The module analysis of co-DEGs using the MCODE plugin found one functional module (Figure 5(c)), and Lox, Timp1, and Spp1 were identified as hub genes.

## 4. Discussion

Cardiac hypertrophy is the cellular response of the heart to physiological stimuli (exercise, pregnancy, etc.) and pathological stimuli (increased pressure or volume load, valvular heart disease, etc.) [1]. Pathological cardiac hypertrophy causes enlargement of cardiomyocytes along the longitudinal axis, upregulation of embryonic gene expression, decreased myocardial contractile function, and irreversible processes such as apoptosis and myocardial fibrosis, which can eventually lead to heart failure [2]. Cardiac hypertrophy is traditionally considered to be a compensatory response to increased workload, to reduce ventricular wall pressure and to maintain cardiac systolic function. However, studies have shown that left ventricular hypertrophy is an independent risk factor for increased cardiovascular morbidity and mortality, even more so than other risk factors. In this experiment, two microarray data related to cardiac hypertrophy in GEO database were mined by bioinformatics, and their common DEGs were screened. Then, the data were analyzed secondarily by biofunctional enrichment, signal pathway enrichment analysis, and protein interaction, in order to provide new therapeutic targets of cardiac hypertrophy.

In the present study, 469 DEGs were screened in GSE1621 and a total of 793 DEGs were screened in GSE18801. GO analyses indicate that DEGs were mainly involved in cardiac muscle contraction, regulation of blood circulation, regulation of muscle contraction, muscle contraction, striated muscle contraction, regulation of heart contraction, regulation of striated muscle contraction, and tissue remodeling. KEGG analyses indicate that DEGs were mainly involved in Th17 cell differentiation, Th1 and Th2 cell differentiation, HIF-1 signaling pathway, pathways in cancer, hematopoietic cell lineage, Chagas disease and cell adhesion molecules, viral myocarditis, central carbon metabolism in cancer, JAK-STAT signaling pathway, and acute myeloid leukemia. HIF-1 is a dimeric transcription factor composed of two subunits, including HIF-1*α* and HIF-1*β*. HIF-1*α* expression induced by pressure load will lead to a significant increase in heart mass to body weight ratio and increased BNP expression and promote the occurrence of myocardial hypertrophy [12]. PI3K is an intracellular phosphatidylinositol kinase. Yu et al. found PI3K could participate in regulating progression of cardiac hypertrophy to heart failure by prolonging autophagic activation, which may be a potential target for the treatment of decompensated myocardial hypertrophy [13]. JAK-STAT is mainly found in cardiomyocytes and is activated by cytokines and growth factors and is a direct signaling pathway linking cell surface receptors to nuclear transcription. Alrasheed et al. suggested simvastatin prevented isoprenaline-induced myocardial hypertrophy by modulating the JAK-STAT signaling pathway [14]. The MAPK signaling pathway is activated by cardiotrophin-1, transforming growth factor-*β*, mechanical stretch, and tyrosine kinase receptors and is involved in a series of protein kinase phosphorylations which is essential in the progression of myocardial pathological hypertrophy [15]. Eight hub genes were screened, including Akt1, Lox, Timp1, Col1al, Spp1, Ccnd1, Mmp3, and Egfr.

Serine/threonine kinase (Akt), also known as PKB, is a direct downstream target protein of PI3K [16]. Akt activation is antiapoptotic and promotes cell proliferation and has roles in regulating cardiomyocyte growth and coronary angiogenesis. Akt is a major effector of PI3K, and only Akt1 and Akt2 of the three Akt genes are expressed in the myocardium. Akt1 knockout mice weighed 20% less than those born in the same litter and had a corresponding reduction in the weight of all tissues, including the heart [17]. The downstream target proteins of the EGFR signaling pathway include AKT, ERK, and STAT3. The EGFR signaling pathway is participated in biological activities like cycle regulation, division, and cell proliferation and is closely associated with the development of cardiovascular diseases such as cardiomyopathy and cardiovascular injury [18]. Kenigsberg et al. found that the EGFR signaling pathway was participated in cell cycle regulation [19]. The cell cycle progresses to the midmitotic phase with the formation of spindle filaments, and microfilaments are formed in large numbers at this stage, resulting in an increase in cell size.

The extracellular matrix (ECM), which surrounds the cardiomyocytes, protects the functional and structural integrity of the heart. Alterations in the extracellular matrix, particularly abnormal alterations in type 1 collagen fibers and type 2 collagen fibers, play an important role in myocardial remodeling. Alterations in the extracellular matrix of the myocardium, particularly the deposition of myocardial collagen that develops asynchronously with cardiomyocyte hypertrophy, are important in contributing to the progression of myocardial hypertrophy. Matrix metalloproteinases (MMPs) of myocardial hypertrophy are protein-cleaving enzymes that hydrolyze the extracellular matrix and consist mainly of collagenases [20]. MMPs can increase abnormal collagen synthesis by degrading the extracellular matrix, stimulating the continuous emergence of new connective tissue and eventually leading to myocardial interstitial fibrosis, ventricular enlargement, and loss of contractile function of the myocardium [21]. MMP3 is a member of the matrix metalloproteinase family that degrades the extracellular matrix and can degrade denatured collagen [22]. In cultured neonatal rat cardiac fibroblasts, TGF-*β*1 upregulated collagen type 1, col1a1, and col3a1. Col1a1 and col3a1 are closely related to the formation of myocardial hypertrophy.

In conclusion, in this study, the DEGs associated with cardiac hypertrophy were screened via bioinformatics analysis, and eight hub genes were identified, including Akt1, Lox, Timp1, Col1al, Spp1, Ccnd1, Mmp3, and Egfr, which might play an important role in the development of cardiac hypertrophy induced by pressure overload, further providing more information for studies on pathogenesis and screening of potential biomarkers of cardiac hypertrophy.

## Data Availability

Data to support the findings of this study is available on reasonable request from the corresponding author.
